# Can Plant Viruses Cross the Kingdom Border and Be Pathogenic to Humans?

**DOI:** 10.3390/v7042074

**Published:** 2015-04-20

**Authors:** Fanny Balique, Hervé Lecoq, Didier Raoult, Philippe Colson

**Affiliations:** 1 Aix-Marseille Université, Unité de Recherche sur les Maladies Infectieuses et Tropicales Émergentes (URMITE) UM 63 CNRS 7278 IRD 3R198 INSERM U1095, Facultés de Médecine et de Pharmacie, 27 boulevard Jean Moulin, 13385 Marseille cedex 05, France; E-Mails: fannybalique@hotmail.fr (F.B.); didier.raoult@gmail.com (D.R.); 2 Institut National de la Recherche Agronomique (INRA), UR 407, Pathologie Végétale, 84140 Montfavet, France; E-Mail: Herve.Lecoq@avignon.inra.fr; 3 Institut Hospitalo-Universitaire (IHU) Méditerranée Infection, Pôle des Maladies Infectieuses et Tropicales Clinique et Biologique, Fédération de Bactériologie-Hygiène-Virologie, Centre Hospitalo-Universitaire Timone, Assistance publique - hôpitaux de Marseille, 264 rue Saint-Pierre, 13385 Marseille cedex 05, France

**Keywords:** plant virus, phytovirus, kingdom, human, animal, pathogenicity

## Abstract

Phytoviruses are highly prevalent in plants worldwide, including vegetables and fruits. Humans, and more generally animals, are exposed daily to these viruses, among which several are extremely stable. It is currently accepted that a strict separation exists between plant and vertebrate viruses regarding their host range and pathogenicity, and plant viruses are believed to infect only plants. Accordingly, plant viruses are not considered to present potential pathogenicity to humans and other vertebrates. Notwithstanding these beliefs, there are many examples where phytoviruses circulate and propagate in insect vectors. Several issues are raised here that question if plant viruses might further cross the kingdom barrier to cause diseases in humans. Indeed, there is close relatedness between some plant and animal viruses, and almost identical gene repertoires. Moreover, plant viruses can be detected in non-human mammals and humans samples, and there are evidence of immune responses to plant viruses in invertebrates, non-human vertebrates and humans, and of the entry of plant viruses or their genomes into non-human mammal cells and bodies after experimental exposure. Overall, the question raised here is unresolved, and several data prompt the additional extensive study of the interactions between phytoviruses and non-human mammals and humans, and the potential of these viruses to cause diseases in humans.

## 1. Introduction

Plant viruses are highly prevalent in plants worldwide, including vegetables and fruits, and represent a serious threat to cultivated plants and agriculture production [1]. Nonetheless, in virology, interest has mainly been focused on viruses that cause diseases in humans and other vertebrates. Thus, among the 833,367 articles found in the NCBI Medline database using “virus” as a keyword, 669,837 articles are found when “virus” is cross-searched with “human” or “animal” compared to 27,148 when “virus” is cross-searched with “plant”. Nevertheless, plant viruses have been seminal in the field of virology. *Tobacco mosaic virus* (TMV), which causes mosaic disease in tobacco plants, is usually quoted as the first virus discovered. Indeed, in 1892, Ivanovski demonstrated that it passed through a Chamberland filter that retains bacteria [2] and in 1898, this experiment was independently reproduced by Beijerinck, who interpreted the result as evidence of a new class of agent [3]. Since that time, multiple plant viruses were discovered and currently they are classified in three orders, 22 families, 108 genera and 1019 species [4]. However metagenomics and high-throughput sequencing have already indicated that plant virus diversity has been underestimated [5,6]. 

**Figure 1:**
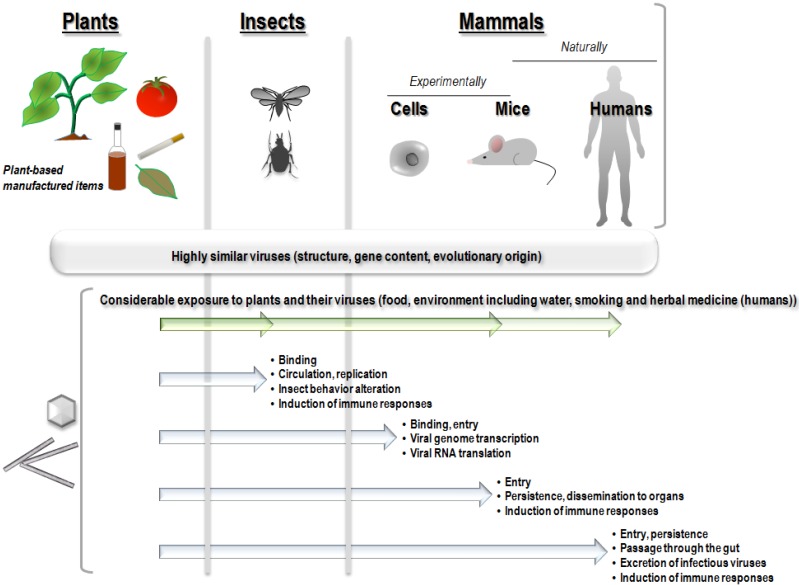
Summary of findings that support border crossing for plant viruses into the invertebrate and vertebrate worlds.

It is currently considered that phytoviruses only infect plants and therefore, plant viruses cannot cause disease in humans. In the present review, several issues are raised that challenge this paradigm (Figure 1).

We describe here that some plant and animal viruses are closely related; humans are considerably exposed to plant viruses; plant viruses can enter mammalian cells and bodies and be naturally present in mammals, including humans; and this presence may be non-neutral; plant viruses may trigger events in mammalian cells and elicit immune responses in invertebrates, non-human vertebrates and humans, and was recently associated with clinical symptoms.

## 2. The Nature of the Border that Segregates Viruses in Plants or Animals

Viruses are obligate intracellular parasites that are capable of infecting eukaryotes, bacteria and archaea, as well as other viruses [7,8,9]. Several differences have been discovered between plant and animal viruses, which have led to a paradigm in which a strict border segregates these viruses either to plants or vertebrates. Certainly, some plant and animal viruses display very dissimilar morphologies and genome structures. The majority of phytoviruses are RNA viruses that often harbor multipartite genomes and have either spherical or rod shapes [10]. In contrast, animal viruses more evenly harbor RNA and DNA genomes, which are mainly monopartite, and a majority of virions have a spherical shape [11]. More practically, major differences exist between plant and animal viruses regarding the mechanisms they use to enter into cells and to then propagate from cell to cell [12]. Thus, specific interactions are implicated between animal viruses and cell receptors before viral entry into the cell by endocytosis or fusion, whereas viral egress from the cell occurs via cell lysis or through budding [13,14]. In contrast, phytoviruses have to cross the rigid plant cell wall composed mainly of cellulose and pectin, and this step does not implicate specific molecular interactions [15]. Thus, phytoviruses can enter plant cells through epidermal cell injuries inflicted by insects that feed on or penetrate plants but also via infected seeds, or through agricultural practices; others are transmitted directly into the phloem, or by whiteflies, nematodes, mites or fungi [15]. Then, plant viruses propagate from cell to cell through the plasmodesmata, a step that requires a movement protein that is only encoded by the genomes of plant viruses [16,17,18].

Notwithstanding these differences, breaking down the kingdom border is far from being unusual for some plant viruses. Indeed, ≈80% of known plant viruses depend on insect vectors for their transmission, although only a small proportion of insect-transmitted viruses are able to replicate in their insect vector [10,12,19]. The majority of these insect vectors are classified into the order *Hemiptera*, which notably encompasses aphids, whiteflies, thrips, leafhoppers and planthoppers [10]. These insects can disseminate viruses to plants and animals thereby creating virus families with wide host ranges. The ability of some plant viruses to infect invertebrate vectors may be related to the presence of a limited number of proteins. For *Potato yellow dwarf virus*, the permissivity of insect cells depends on the presence of a glycosylated protein G [20], and for *Rice dwarf virus* a mutation of the P2 protein may abolish virus infectivity in the insect, and its transmission capacity [21]. There are two main modes of plant virus transmission by insects: non-circulative transmission, where the virus remains attached to the cuticle of the insect stylet or the anterior digestive tract of the insect vector but does not cross insect body barriers, and circulative transmission, where the virus crosses insect body barriers and enters its circulatory system and then accumulates in the salivary glands [12,22]. This specific viral adhesion to the cuticle can involve either capsid proteins that allow adhesion to insect receptors, as for *Cucumber mosaic virus* (CMV) [23], or through other viral proteins called helper components. In the case of potyviruses, one conserved domain (PTK) of the helper component protein (HC) has been shown to interact with a conserved capsid domain (DAG), and another conserved domain of HC is believed to interact with hypothetical receptors on the insect stylet [12]. In circulative transmission, infected saliva transmits the virus to healthy plants via insect feeding. In circulative non-propagative transmission, viruses only transit through the insect body but do not replicate. Members of the families *Luteoviridae*, *Nanoviridae* and *Geminiviridae* are transmitted to plants via this mode. *Tomato yellow leaf curl virus* (TYLCV), which belongs to family *Geminiviridae*, is suspected to multiply in its insect vector, *Bemisia tabaci*, and transovarial and sexual transmission of this virus and its DNA have been observed [24,25,26]. In circulative propagative transmission, viruses multiply in the insect vector and can be found in different organs and tissue such as muscle, nervous tissue, connective tissue, salivary glands and fat, and transovarial transmission has been described for some viruses [27]. Viruses transmitted via the circulative propagative mode include members of the family *Rhabdoviridae* (genera *Cytorhabdovirus* and *Nucleorhabdovirus*), *Reoviridae* (genera *Phytoreovirus*, *Fijivirus* and *Oryzavirus*), *Tymoviridae* (genus *Marafivirus*), *Bunyaviridae* (genus *Tospovirus*), and the genus *Tenuivirus* [10]. The fact that some plant viruses can replicate in their insect vectors makes them somewhat insect viruses as well, and phytoviruses involved in circulative propagative transmission are generally considered to be insect viruses that have acquired the ability to infect another reservoir, namely plants, during evolution. Moreover, in insects, alterations in behavior have been observed in association with infection by several plant viruses, suggesting that these viruses may be pathogenic for the insects (Figure 1). *Tomato spotted wilt virus* (TSWV) has been shown to directly alter the male feeding behavior of *Frankliniella occidentalis* [28]. In addition, infection of *Bemisia tabaci* by TYLCV was associated with a reduction in insects of longevity and fecundity [29], and recently, *Tobacco ringspot virus* (TRSV), a pollen-borne nepovirus, was shown to replicate and produce virions in several body parts in *Apis mellifera*, honeybees [30].

**Figure 2:**
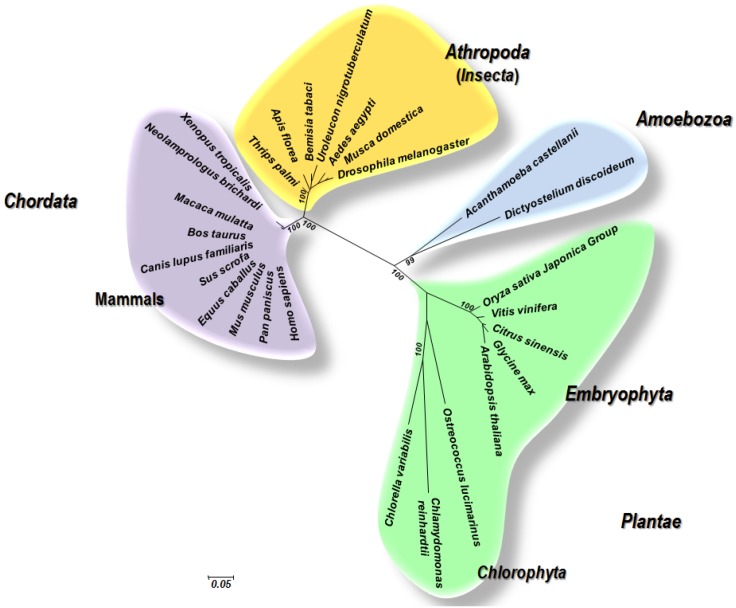
Phylogeny reconstruction based DNA-dependent RNA polymerase subunit 2 for some members of the domain Eukaryota, including land plants, insects and human and other mammals. Phylogenetic analysis was performed using the maximum likelihood method with the MEGA5 software [33], and involved a curated alignment of 27 sequences composed of 1038 amino acid positions. Only bootstrap values at major nodes are shown. Scale bar represents the number of estimated changes per position for a unit of branch length. Sequences from the NCBI GenBank protein sequence database [34] that were used included: CAA79527, *Arabidopsis thaliana*; XP_006487405, *Citrus sinensis*; XP_636812, *Dictyostelium discoideum* AX4; EEE59590, *Oryza sativa* Japonica Group; XP_005844289, *Chlorella variabilis*; XP_003547482, *Glycine max*; BAJ78717, *Bemisia tabaci*; XP_002274051, *Vitis vinifera*; EJY57456, *Aedes aegypti*; XP_003129085, *Sus scrofa*; CAA29180, *Drosophila melanogaster*; XP_001415447, *Ostreococcus lucimarinus* CCE9901; XP_002937598, *Xenopus (Silurana) tropicalis*; AAH38472, *Mus musculus*; XP_001916826, *Equus caballus*; NP_000929, *Homo sapiens*; XP_003806546, *Pan paniscus*; XP_006781988, *Neolamprologus brichardi*; XP_532382, *Canis lupus familiaris*; XP_005188839, *Musca domestica*; EHH25986, *Macaca mulatta*; XP_003696177, *Apis florea*; BAJ78718, *Uroleucon nigrotuberculatum*; XP_004348530, *Acanthamoeba castellanii* str. Neff; BAJ78713, *Thrips palmi*; NP_001092552, *Bos taurus*; AAY89365, *Chlamydomonas reinhardtii*.

These previous findings question if some plant/insect viruses may further cross the border into the animal world to enter and replicate in vertebrates. In fact, with respect to the phylogenetic distance between plants on the one hand and insects or mammals including humans on the other hand, it appears just as complex for viruses to cross the kingdom barrier from plants to insects than from plants to any mammals. Indeed, phylogenetic distance between plants and insects, which are natural and possible hosts for some plant viruses, is far greater than the distance between insects and vertebrates (Figure 2) [31,32].

## 3. Close Relatedness between Plant and Animal Viruses and Evidence for Common Origins

The distribution of plant and animal viruses within the currently defined taxons indicates that some plant and animal viruses belong to same viral families (Table 1).

Indeed, family *Reoviridae* includes several genera infecting a variety of animals and three genera (*Phytoreovirus*, *Fijivirus* and *Oryzavirus*) infecting both plants and insects [11,35]. In addition, most of the phytoviruses that multiply in insect vectors and are transmitted through the circulative mode are classified in families that also contain animal viruses. This is true for family *Reoviridae*, which includes rotavirus, a major cause of gastroenteritis in humans [36], family *Rhabdoviridae*, which notably includes rabies virus [37], and family *Bunyaviridae*, which encompasses human pathogens such as Hantaan virus and Toscana virus [38,39,40]. Within the family *Rhabdoviridae*, genomic organization indicates that some plant viruses of genera *Nucleorhabdovirus* and *Cytorhabdoviru*s differ from animal viruses of genera *Vesiculovirus*, *Lyssavirus*, *Novirhabdovirus* and *Ephemerovirus* only by the presence of an additional gene coding for a movement protein, a protein essential for the systemic spread of the virus in the host plant [17,18,37,41]. In addition among the genera of animal rhabdoviruses, many viruses are characterized as arboviruses and can be transmitted by insects vectors. The *Vesicular stomatitis virus* (VSV) which belongs to genus *Vesiculovirus*, can be transmitted to domestic swine through infected black-flies (*Simulium vittatum*) [42]. Notably, reoviruses, rhabdoviruses and bunyaviruses, in addition to containing members infecting either plants or insects or vertebrates, all have virions with an envelope derived from the host membrane. This, together with membrane-inserted viral-encoded proteins that interact with cell surface receptors, facilitates entry into animal host cells.

**Table 1:** Virus families composed of plant and humans or non-human animal viruses.

Genome	Genome Segmentation	Virion Shape	Family	Sub-Family	Genus	Hosts
RNA, single-stranded, positive	Monopartite	Icosahedral	*Tymoviridae*		*Tymovirus*	Plants
	Monopartite				*Maculavirus*	Plants
	Monopartite				*Marafivirus*	Plants and insects
RNA, single-stranded, negative or	Monopartite	Pleomorphic, globular	*Bunyaviridae*		*Hantavirus*	Mammals
ambisense	Monopartite				*Bunyavirus*	Mammals
	Monopartite				*Nairovirus*	Mammals
	Monopartite				*Phlebovirus*	Mammals
	Tripartite				*Tospovirus*	Plants and insects
RNA, single-stranded negative	Monopartite	bacilliform	*Rhabdoviridae*		*Lyssavirus*	Vertebrates
	Monopartite				*Vesiculovirus*	Vertebrates
	Monopartite				*Ephemerovirus*	Vertebrates
	Monopartite				*Novirhabdovirus*	Vertebrates (fishes)
	Monopartite				*Cytorhabdovirus*	Plants and insects
	Monopartite				*Nucleorhabdovirus*	Plants and insects
	Bipartite				*Dichorhabdovirus*	Plants
	Multipartite	Flexuous	*-*		*Tenuivirus*	Plants and insects
RNA, double-stranded	Monopartite	icosahedral	*Reoviridae*	*Sedoreovirinae*	*Cardoreovirus*	Crustaceans
	Monopartite				*Mimoreovirus*	Photosynthetic marine protists
	Monopartite				*Orbivirus*	Arthropod, vertebrates
	Monopartite				*Rotavirus*	Vertebrates
	Monopartite				*Seadornavirus*	Invertebrates and vertebrates
	Multipartite				*Phytoreovirus*	Plants and insects
	Monopartite			*Spinareovirinae*	*Aquareovirus*	Aquatic vertebrates and invertebrates
	Monopartite				*Coltivirus*	Invertebrates and vertebrates
	Monopartite				*Cypovirus*	Insects
	Monopartite				*Dinovernavirus*	Insects
	Monopartite				*Idnoreovirus*	Insects
	Monopartite				*Mycoreovirus*	Fungi
	Monopartite				*Orthoreovirus*	Vertebrates
	Multipartite				*Fijivirus*	Plants and insects
	Multipartite				*Oryzavirus*	Plants and insects

In addition, *Cowpea mosaic virus* (CPMV), which is a member of family *Secoviridae*, shares structural features and genetic organization with animal picornaviruses such as polioviruses, coxsackie viruses and *Theiler’s murine encephalomyelitis virus* (TMEV), which supports the hypothesis that these plant and animal viruses might derive from a common ancestor [43,44]. In addition, pararetroviruses include plant viruses from the family *Caulimoviridae*, which consists of the only plant viruses with a double-stranded (ds) DNA genome, as well as animal viruses that belong to the family *Hepadnaviridae*, which includes hepatitis B virus [45]. Similarly, hepatitis E virus, a leading cause of acute hepatitis in humans, was grouped with *Beet necrotic yellow vein virus*, a plant virus that belongs to the genus *Benyvirus*, based on phylogeny of RNA polymerases [46]. Recently, endogenous viral elements (EVEs) from plant RNA viruses were detected in 14 insect genomes [47]. Interestingly, EVE sequences very close to those of phytoviruses of the family *Virgaviridae* were detected in the genome of a mosquito, *Aedes aegypti*; a fly, *Drosophila rhopaloa*; and a bee, *Bombyx terrestris*. The EVE detected in *Aedes aegypti* was similar to the tobamovirus genome, which is very surprising as no insect vector is known for tobamoviruses. These insects might have possibly interacted with these viruses during nectar feeding on infected plants. Moreover, Dolja and Koonin noted that there are multiple related groups of viruses in plant and animals and that the genes involved in viral genome replication and expression are conserved between plant and animal viruses, whereas genes implicated in virus-host interactions are not [48]. These authors described three scenarios for the evolution of related viruses in plants and animals: (i) evolution of these viruses from a common ancestral virus predating plant-animal divergence; (ii) horizontal transfers of viruses between plants and animals; and (iii) parallel evolution of related viruses from related ancestral genetic elements [48]. For instance, RNA-dependent RNA polymerase (RdRp) of positive single-stranded as well as dsRNA viruses that infect plants or animals have conserved amino acid sequences and three-dimensional structures, which suggests common roots for these viruses [46,48,49]. In addition, the rolling-circle replication endonuclease (RCRE), a key enzyme for the replication of single-stranded DNA viruses, is conserved in plant viruses (families *Geminiviridae* and *Nanoviridae*) and in animal viruses (families *Parvoviridae* and *Circoviridae*) [48,50,51]. Also, Gibbs *et al.* suggested that a plant nanovirus switched hosts to infect a vertebrate and then recombined with a vertebrate-infecting picorna-like virus [52,53]. This host-switch might have occurred through exposure of a vertebrate to the sap of an infected plant. Lastly, the delta agent, typically called *hepatitis delta virus* (HDV), which is a human pathogen that needs the envelope protein of *hepatitis B virus* for its replication, displays considerable similarities with viroids [59]. Viroids are plant pathogens smaller and simpler than plant viruses, only composed of a naked single-stranded circular non-coding RNA of ≈250–400 nucleotides with a rod-like secondary structure due to self-complementary regions [54]. Regarding the HDV genome, it is 1700 nucleotide-long, which is the smallest size among known animal viruses, and it encodes a single protein (the delta antigen) [55,56]. This genome is a covalently closed circular single-stranded molecule that might have a rod-like secondary structure, as approximately two-thirds of the bases are paired [55], and it has been hypothesized that HDV may have evolved from the fusion of a viroid with an animal gene [57,58]. Indeed, Brazas and Ganem isolated and cloned a cellular gene that encodes a protein named delta-interacting protein A (DIPA), which shares 56% amino acid similarity with the delta antigen [57]. These authors hypothesized that DIPA and HDV antigen-encoding RNA may share a common ancestor that could have been captured by a viroid-like RNA. 

## 4. Exposure of Non-Human Animals and Humans to Plant Viruses

Non-human animals and humans that eat fruits and leafy vegetables are exposed to phytoviruses. Thus, plant viruses are most likely highly prevalent in wild and cultivated plants, including fruits and vegetables (Figure 1). For example, it was found that approximately 60% of plants in a geographical area of Costa Rica (encompassing a total of 7000 plant species) harbored plant viruses [5]. TMV may infect over 150 plants including tomatoes, peppers, and cucumbers [59]. *Tomato bushy stunt virus* (TBSV), a tombusvirus, is an important pathogen of tomatoes and other plants, and it was observed to reach a concentration of approximately 200 mg per kg of infected leaves in experimental hosts such as *Nicotiana clevelandii* [60]. Moreover, this virus can remain infectious despite being frozen for several years, and its thermal inactivation point is 80–90 °C [61]. Some phytoviruses are also present in food products, and we detected *Pepper mild mottle virus* (PMMoV) RNA in 57% of 28 pepper-based food items and demonstrated that the PMMoV in these food items was still able to induce plant infection [62]. The viral titer can be very high in these products. For example, we detected up to 10^7^ viral copies/mL in Tabasco sauce [62]. Hence, it can be anticipated that plant viruses could be found in human faeces. Humans can also be exposed to plant viruses through ingestion of herbal medicine. In some countries in Asia and sub-Saharan Africa, 80% of the population uses traditional medicine for primary health care, with herbal treatment being the most used traditional medicine [63]. Smoking is another risk factor for exposure to plant viruses, TMV being present and stable in the smoked tobacco [64,65,66,67] and resistant to manufacturing processes [68]. Mean TMV RNA titer was found to be 9.5 log_10_RNA copies/cigarette and 3.8 log_10_RNA copies/mL of smokers’ saliva [69].

Finally, plant viruses are also detected in the environment including in the soil, water, and clouds [70,71,72]. ToMV was found in water draining in forest stands in New York State [73], in 140,000-year-old glacial ice from drill sites in Greenland [74], and also in fogs and clouds [75]. PMMoV has been found to be widespread and abundant in wastewater in the United States and in sewage and river water in Germany, which suggests that this virus can be a good indicator of human fecal pollution [76,77], and it was recently detected in 76% of 184 source water samples collected from 30 drinking water treatment plants in Japan between 2008–2011, at concentrations up to 3.5 log_10_copies/mL [78]. In addition, TBSV was recovered from the Thames river and several other English rivers [79], ToMV and *Melon necrotic spot virus* have been detected in irrigation systems in Slovenia and Spain, respectively [80,81], and *Cucumber green mottle mosaic virus* (CGMMV) was recovered from the Yamuna River in India, and the recovered viruses were infectious to host plants [82]. This capability to persist in the environment enhances the dispersion and transmission of these viruses.

## 5. Evidence of the Natural Presence and Persistence of Plant Viruses in Non-Human Mammals

**Figure 3:**
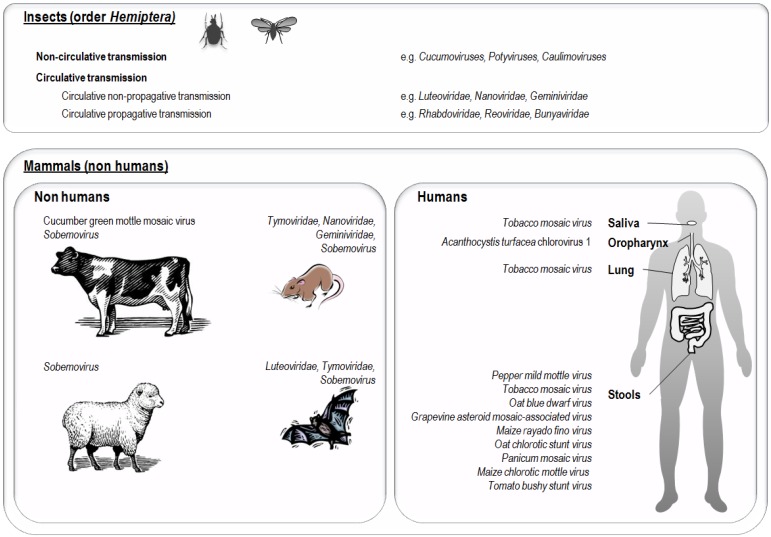
Detection of plant viruses in insects and mammals including humans.

The natural presence of plant viruses in non-human mammals has been reported in several studies (Table 2; Figure 1 and Figure 3), and these mammals may spread infectious phytoviruses that are stable in the gastrointestinal tract through their contaminated stools [83,84].

**Table 2:** Plant viruses detected in humans or non-human mammals or shown to interact in experimental *in vitro* studies with humans or non-human mammals.

Nucleic Acid	Family	Genus	Species	Vertebrate Cells	Mammals	Humans
ssRNA+	*Alphaflexiviridae*	N.a.			Mouse, vole and rat stools [85]	
ssRNA+	*Bromoviridae*	*Ilarvirus*	*Prunus necrotic ringspot virus*			Stools [86]
ssRNA+	*Closteroviridae*	*Closterovirus*	*Citrus tristeza virus*			Stools [87]
ssRNA+	*Luteoviridae*	N.a.	N.a.		Bat guano [88]	
		N.a.	N.a.		Mouse, vole and rat stools [85]	
ssRNA+	*Secoviridae*	*Comovirus*	*Cowpea mosaic virus*	Human Hela cells [44]	Mice [84]	
		*Comovirus*	*Cowpea mosaic virus*	Human huvec cells [44]		
		*Comovirus*	*Cowpea mosaic virus*	Human KB cells [44]		
		N.a.	N.a.		Bat guano [88]	
		N.a.	N.a.		Mouse, vole and rat stools [85]	
ssRNA+		*Sobemovirus*	*Rice yellow mottle virus*		Cows, donkeys and grass rats [89]	
		*Sobemovirus*	*Subterranean clover mottle virus*		Sheep [90]	
		*Sobemovirus*	N.a		Bat guano [88]	
		*Sobemovirus*	*Cocksfoot mottle virus*			Stools [86]
ssRNA+	*Tombusviridae*	*Tombusvirus*	*Tomato bushy stunt virus*			Stools [60]
		*Panicovirus*	*Panicum mosaic virus*			Stools [86]
		*Carmovirus*	*Melon necrotic spot virus*			Stools [86]
		*Carmovirus*	*Galinsoga mosaic virus*			Stools [86]
		*Carmovirus*	*Carnation mottle virus*			Stools [86]
		*Necrovirus*	*Tobacco necrosis virus*			Stools [86]
		*Necrovirus*	*Olive latent virus 1*			Stools [86]
		*Aureusvirus*	*Pothos latent virus*			Stools [86]
		*Aureusvirus*	*Johnsongrass chlorotic stripe mosaic virus*			Stools [86]
		*Avenavirus*	*Oat chlorotic stunt virus*			Stools [86]
		*Machlomovirus*	*Maize chlorotic mottle virus*			Stools [86]
		N.a.	N.a.		Mouse, vole and rat stools [85]	
ssRNA+	*Tymoviridae*	*Marafivirus*	*Grapevine asteroid mosaic-associated virus*			Stools [86]
		*Marafivirus*	*Maize rayado fino virus*			Stools [86]
		*Marafivirus*	*Grapevine rupestris vein feathering virus*			Stools [86]
		*Marafivirus*	*Oat blue dwarf virus*			Stools [86]
		*Maculavirus*	*Grapevine fleck virus*			Stools [86]
		*Maculavirus*	*Grapevine red globe virus*			Stools [86]
		*Tymovirus*	*Chayote mosaic tymovirus*			Stools [86]
		*Tymovirus*	*Kennedya yellow mosaic virus*			Stools [86]
		*Tymovirus*	*Physalis mottle virus*			Stools [86]
		*Tymovirus*	*Poinsettia mosaic virus*			Stools [86]
		*Tymovirus*	*Eggplant mosaic virus*			Stools [86]
		*Tymovirus*	*Ononis yellow mosaic virus*			Stools [86]
		N.a.	N.a.		Bat guano [88]	
		N.a.	N.a.		Mouse, vole and rat stools [85]	
ssRNA+	*Virgariridae*	*Tobamovirus*	*Tobacco mosaic virus*	Hela cells [91]	Mice [92]	Stools [86,87]
				Mice splenocyte [93]		Skin [94]
				Mice bone marrow cells [92]		Thoracentesis fluids [95]
						Saliva [69]
		*Tobamovirus*	*Pepper mild mottle Virus*			Stools [86,87]
		*Tobamovirus*	*Cucumber green mottle mosaic virus*		Cows stools [96]	
		*Tobamovirus*	*Tomato mosaic virus*			Stools [86]
		*Tobamovirus*	*Ribgrass mosaic virus*			Stools [86]
		*Tobamovirus*	*Turnip vein-clearing virus*			Stools [86]
		*Tobamovirus*	*Tobacco mild green mosaic virus*			Stools [86]
		*Tobamovirus*	*Odontoglossum ringspot virus*			Stools [86]
		*Tobamovirus*	*Paprika mild mottle virus*			Stools [86]
		*Tobamovirus*	*Kyuri green mottle mosaic virus*			Stools [87]
		*Tobamovirus*	*Crucifer tobamovirus*			Stools [87]
		*Tobamovirus*	*Obuda pepper virus*			Stools [86]
		*Tobamovirus*	*Nigerian tobacco latent virus*			Stools [86]
dsRNA	*Partitiviridae*	N.a.	N.a.		Bat guano [88]	
		N.a.	N.a.		Mouse, vole and rat stools [85]	
dsDNA	*Phycodnaviridae*	*Chlorovirus*	*Acanthocystis turfacea* chlorella virus 1		Mouse [72]	Oropharynx samples [72]
ssDNA	*Nanoviridae*	N.a.	N.a.		Mouse, vole and rat stools [85]	
ssDNA	*Geminiviridae*	N.a.	N.a.		Mouse, vole and rat stools [85]	

In 1988, CGMMV, a tobamovirus, was recovered in cow manure three days after feeding with infected cucumbers [96]. More recently, 46% of sequences from a Californian bat guano virome and 27% of a Texan bat guano virome were linked to members of *Luteoviridae* and *Sobemovirus* [88]. In the stools of wild rodents, 3.4% of virome sequences were linked to phytoviruses of families *Nanoviridae*, *Geminiviridae*, and *Alphaflexiviridae* [85]. Members of the families *Partitiviridae*, *Tymoviridae* and *Secoviridae* were also observed in bat and rodent stools [85,88]. PMMoV was detected by qRT-PCR in 7/15 fecal samples from chicken, 1/10 from geese and 1/6 from cows, at titers up to 3.1 log_10_copies/mg [97]. In addition, the transmission of sobemoviruses by animals was also reported; the *Rice yellow mottle virus* can be transmitted by cows, donkeys and grass rats [89], and the *Subterranean clover mottle virus* by sheep (Table 2) [90].

## 6. Presence of Plant Viruses in Human Samples

In 1982, TBSV, a *Tombusviridae* member, was purified and ingested by human volunteers. The inoculation of their feces on *Chenopodium quinoa* leaves induced signs of viral infection. It was also hypothesized that TBSV could be spread by humans in river water through sewage [60,79]. Moreover, several studies conducted during the 1950s–1970s reported on the presence of TMV in human lungs and its role in lung cancer [64,98,99,100]. Thus, TMV was recovered from lung cancerous matter [94] (Figure 3), from 15/35 sputum specimens and 1/4 thoracentesis fluids from cigarette smokers with a history of pulmonary disease [95], but another study failed to confirm this hypothesis [101]. TMV was also detected in the lungs of active or passive smokers through inoculation to tobacco leaves [102,103]. Nevertheless, this ability for a very stable virus such as TMV to retain infectivity does not demonstrate that it replicated within a smoker’s respiratory fluids or organs. Also, tobacco plant DNA was recently detected in the bronchoalveolar lavage of intubated patients [104]. The issue of plant virus presence and pathogenicity in humans was fueled in 2006 by the discovery by metagenomics that plant viruses were the most prevalent RNA viruses in human feces (Table 2; Figure 3) [86]. The most abundant virus was PMMoV, but this study identified 35 phytoviruses including TMV. Up to 10^9^ virions of PMMoV were detected per gram of dry weight of fecal matter. We sought PMMoV using real-time PCR in feces and detected it from 22 (7.2%) of 304 adults [62]. We also observed a correlation between PMMoV presence in stools and some clinical signs. Indeed, fever was observed in 39% of PMMoV-positive *versus* 13% of PMMoV-negative patients (*p* *=* 0.042), and abdominal pain were reported in 39% of PMMoV-positive *versus* 7% of PMMoV-negative patients (*p* = 0.008), and pruritus was also independently correlated with PMMoV RNA detection in feces in multivariate analysis. Nevertheless, no causal relationship was demonstrated between PMMoV presence and clinical symptoms; especially, abdominal pain may be related to the consumption of pepper or pepper-based food items, which was not assessed in our study. Nonetheless, taken together, these findings support the hypothesis that PMMoV may not be only a transient inhabitant of the human gut but, alternatively, its presence may be non-neutral. In another study, PMMoV was found by qRT-PCR in 19/20 healthy human fecal samples at titers up to 7.0 log_10_viral RNA copies/mg [97]. In addition, using metagenomics, tobamoviruses were detected in stools from Japan (Table 2; Figure 3) [87] and TMV RNA was retrieved in stools collected from South Asian children with non-polio acute flaccid paralysis [105], concurrently with human pathogens, which were presumed to be responsible for the observed symptoms. We also detected TMV RNA in 45% of saliva samples from smokers whereas all saliva samples from non-smokers tested negative, and we found that all 47 cigarettes from different brands were positive for TMV (Figure 1) [69]. 

Finally, two recent studies described the presence of algae viruses in humans. Stepanova *et al.* reported such viruses by culturing on the marine diatom *Phaeodactylum tricornutum* in 16 of 41 (39%) pooled cervicovaginal secretions from women from Ukraine presenting with colpitis (6 of 11), uterus fibroids (5 of 14), or uterus cervical erosion (5 of 16) [71]. More strikingly, it was reported in 2014 that many metagenomic sequences retrieved from human oropharyngeal samples matched to *Acanthocystis turfacea* chlorella virus 1 (ATCV-1), a phycodnavirus that infects green algae [72]. Moreover, ATCV-1-liked DNA was subsequently detected from 44% of 92 oropharyngeal samples by real-time PCR, and PCR positivity was significantly correlated with altered cognitive functions. ATCV-1 inoculation to mice also impaired cognitive functions, and significantly altered hippocampus gene expression, as assessed 26 weeks post-exposure.

## 7. Immune Responses to Plant Viruses in Invertebrates, Non-Human Vertebrates and Humans

Plant viruses were found to trigger immune responses in invertebrates, vertebrates and humans, which might only reflect a history of exposure to foreign proteins and does not necessarily imply a role of the plant virus in pathogenesis. TSWV infection has been shown to induce a strong immune response in *Frankliniella occidentalis*, its major insect vector [106]. In addition, CPMV has been shown to induce humoral and persistent systemic and local immune responses in mice as detected by ELISA following oral administration [107], mice inoculated with *Potato Virus Y* developed antibodies to the virus [108], and we detected anti-TMV total antibodies in serum samples from mice inoculated intratracheally with TMV, seven days after inoculation [92]. In another work, inoculation to mice of ATCV-1, a phycodnavirus that infects green algae, was associated with the appearance of antibodies to this virus in 36% of the mice [72]. Finally, we found anti-PMMoV antibodies in humans, significantly more frequently in serum samples from patients with PMMoV, and, than in serum samples from controls [62], and anti-TMV IgG were recently detected by another team at a higher level in smokers than in non-smokers [109].

## 8. Evidence of the Entry of Plant Viruses or Their Genomes into Non-Human Mammal Cells and Bodies after Experimental Exposure

Experimental data are available on the entry of plant viruses or their genomes into non-human mammal cells and bodies, although this was obtained through artificial means and did not lead to viral propagation in absence of an exogenous host factor. Indeed, viral replication implies the formation and release of a viral progeny, following virus entry in the target host cells. *In vitro* analyses have demonstrated that TMV RNA can be translated in *Xenopus laevis* oocytes [110] (Figure 1). TMV was used to model the defense mechanisms of HeLa cells against virus invasion, which allowed detecting an accumulation of TMV proteins on autophagosomal membranes, and observing TMV-resembling virions in the cellular cytoplasm [91]. In addition, we recently observed that infectious TMV could enter and persist in mouse lungs inoculated via the intratracheal route, as determined using immunohistochemistry, electron microscopy, real-time RT-PCR and sequencing; anti-TMV antibody seroconversions were observed in mice and TMV was detected in murine bone marrow-derived macrophages following experimental inoculation [92]. Moreover, in 2009, Koudelka *et al.* demonstrated that CPMV binds to the vimentin surface protein and enters mammalian cells, including endothelial, circulating cells and HeLa cells [44]. The animal picornavirus TMEV has also been shown to interact directly with vimentin [111], and mammalian *Porcine reproductive and respiratory syndrome virus* also uses surface vimentin for entry in host cells [112]. Thus, CPMV and animal viruses share the processes of entry into mammalian cells, which supports the possibility of cross-kingdom transmission [44]. RNA of *Flock house virus* (FHV), an insect virus first isolated from the grass grub *Costelytra zealandica* that belongs to the family *Nodaviridae*, whose members include viruses infecting various invertebrates and vertebrates, has been reported to replicate in plant, insect, yeast and mammalian (baby hamster kidney) cells, and it has been hypothesized that the host components required for FHV replication could be widely conserved [113,114,115,116,117,118,119]. In addition, CPMV has been shown to resist and remain infectious to plants after exposure to acid pH and low concentrations of pepsin [83] and treatment with simulated gastric fluid-containing pepsin at pH 2 or simulated intestinal fluid-containing pancreatin at pH 6.8, suggesting stability for this virus in the gastrointestinal tract [84]. CPMV was demonstrated to be capable of entering the bloodstream and disseminating through systemic trafficking [84], as the presence of the virus was observed in many different organs, such as the spleen, kidney, lung, stomach, duodenum, jejunum, ileum, lymph node, brain, bone marrow and blood, over three days following mouse inoculation. Although replication of a plant virus in mammalian cells was not demonstrated without requiring expression of a genuine host protein, a study reported in 2005 that overexpression of *Frankliniella occidentalis* encoded putative transcription factors in two human cell lines, human epithelial carcinoma (HeLa) cells and diploid fibroblasts, made these cells permissive for the replication of TWSV [120].

## 9. Conclusions

Previous findings suggest that the border between plants and mammals including humans may be less strict for plant viruses than currently described (Figure 1). Mankind has likely been considerably exposed to plant viruses for several thousand years, and this supports the paradigm that these viruses are safe for humans. Alternatively, if a plant virus was to completely breakdown the host specificity border and become able to multiply in vertebrates, this multiplication could remain unnoticed if it is not associated with a specific symptomatology and could also remain an evolutionary dead-end for the virus if there is no further transmission to vertebrates. Certainly, no evidence that plant viruses are causative agents of disease in humans and other animals has been revealed to date. A very limited number of published studies have been designed to address the issue of the potential pathogenicity of phytoviruses in humans. On the contrary, modified plant viruses, such as TMV, PVX, CMV, PapMV and CPMV have been used recently in new vaccine approaches [121,122,123,124,125,126,127,128]. Overall, previous data indicate that the role of plant viruses as potential human pathogens deserves to be specifically studied further. In particular, this possible role may be deciphered by studying whether and how plant viruses may interact with human cells, which might not necessarily be associated with viral replication in permissive host cells but might involve modulation of gene expression in human cells through RNA interference mechanisms [129].
